# A functional systems view on neural tracking of natural speech

**DOI:** 10.3389/fnsys.2025.1658243

**Published:** 2025-09-03

**Authors:** Anton Rogachev, Olga Sysoeva

**Affiliations:** ^1^Center for Cognitive Sciences, Sirius University of Science and Technology, Sirius, Russia; ^2^Laboratory of Higher Nervous Activity of Human, Institute of Higher Nervous Activity and Neurophysiology, Russian Academy of Sciences, Moscow, Russia

**Keywords:** neural tracking, neural entrainment, natural speech, functional systems, brain activity, rhythmic activity

## 1 Introduction

Speech is a core component of human life underlying communication with the world and inner mental life. For many years, human ability to comprehend and produce speech has been widely studied in psychology and neuroscience. Neuroscience uses a wide range of invasive and non-invasive neuroimaging techniques to study brain responses to acoustic, phonetic, lexical and semantic features of speech, and how the speech acoustic (physical signal) is mapped to words, sentences and texts meanings (non-material semantics) through cerebral cortex.

An important way of neuroscience of speech research in the last decade is the focus on naturalistic speech stimuli (Hamilton and Huth, 2020; Zoefel and Kösem, 2024). In this context, studies of *neural tracking of natural speech* have a special place in literature. Neural tracking, or neural entrainment, could be defined as a phenomenon of synchronization between brain electrical activity and continuous or discrete changes in the speech stimulus components during its active, attentive perception (Lalor and Foxe, 2010; Ding and Simon, 2014). Many studies revealed that neural tracking is functionally related to speech comprehension (Ahissar et al., 2001; Broderick et al., 2022), the level of attention directed to speech (Crosse et al., 2016; Sánchez-Costa et al., 2025), and the development of speech and language in children (Rogachev and Sysoeva, 2024). Moreover, neural tracking is disturbed in some clinical cases of developmental language disorders (Nora et al., 2024) or post-stroke aphasia (De Clercq et al., 2025). Thus, neural tracking research has both fundamental and applied significance and potential implications.

In the literature, there is a substantial discussion on the nature and functional roles of neural tracking of speech. Summarizing, this discussion raises several questions. What is neural tracking physiologically: endogenous oscillatory activity associated with processing the rhythm of speech, or the sum of evoked activity on the acoustic edges of a speech audio signal (Meyer et al., 2020; Lalor and Nidiffer, 2025)? Are the so-called speech rhythms [e.g., electromagnetic rhythmic brain activity related to the averaged rhythms of phonemes, words and phrases in a natural speech (Poeppel and Assaneo, 2020)] indeed linked to the rhythms of speech through endogenous oscillators tuned to the rhythm of the native language in ontogenesis, or are they also only represent evoked activity (Kazanina and Tavano, 2023a,b)? What is the contribution of top-down processes, i.e., the influence of prior experience and predictions about speech content, on neural tracking (Broderick et al., 2019; Gwilliams, 2020; Klimovich-Gray and Molinaro, 2020; Klimovich-Gray et al., 2023)?

We propose that these questions can be narrowed to the one: is neural tracking an active process, based on endogenous oscillators activity which is based on their inner state and predictions about speech content, or is it a passive process of cortical tuning to the rhythm of speech stimulus? Of course, the answer to this question requires a lot of empirical work. However, an attempt to address this question can be given from a theory-driven point of view, which may provide a new perspective on the problem of the nature of neural tracking. Here, we provide a possible theoretical interpretation of neural tracking of speech from the perspective of functional systems theory to raise questions regarding the construction of experiments on natural speech perception and data analysis approaches, empirical answers to that can help solve common neural tracking issues.

## 2 Functional systems theory

Functional systems theory (FST) is a psychophysiological theoretical framework, developed by Russian and Soviet physiologist Pyotr Anokhin, describing the mechanisms of interaction between a person (and, in general, any organism) and the external environment (Anokhin, 1971; Egiazaryan and Sudakov, 2007; Rusalov, 2018; Vityaev and Demin, 2018; Shadrikov, 2019). Functional system (FS), a core concept of FST, is the dynamic integration of different body and brain systems for the realization of behavior aimed at achieving a specific adaptive goal. For example, if an organism begins to experience hunger, its physiological systems (sensory, central and motor) will be tuned to solve a sequence of tasks: searching for opportunities to get food, deciding on a specific search strategy, performing these actions, etc., with possible correction of decisions during the execution. According to FST, any organism is an active subject whose behavior is determined by an adaptive behavioral goal in the future rather than a set of environmental or intrinsic stimuli in the past.

**Figure 1:**
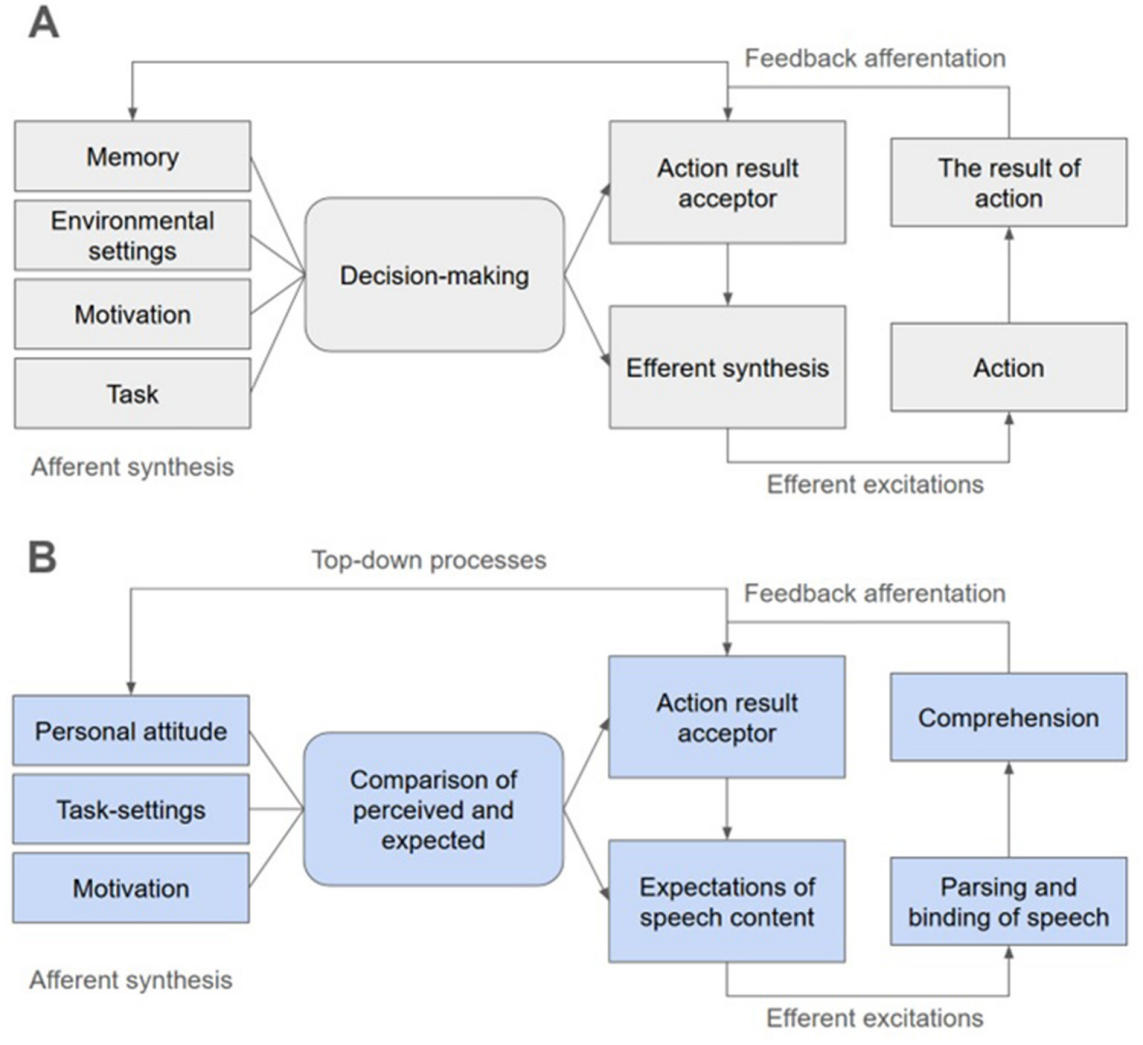
**(A)** The typical structure of a functional system according to Anokhin's functional systems theory. **(B)** A potential interpretation of the process of neural tracking of natural speech in the context of functional systems theory.

The typical structure of FS is presented in Figure 1A. In FS, there are four units connected by feedback loops. The first unit, afferent synthesis, synthesizes information preceding a current behavioral act (i.e., motivation, previous experience, external environment state, task specifications, etc.). Based on afferent synthesis, a decision is made, which is also based on the image of the result, which is located in the action result acceptor. This resulted in efferent synthesis, in which mental and somatic systems are programmed to perform a behavioral act to achieve the result. Next, the behavioral act itself is realized. During its realization, there is a constant comparison of current results with expected ones by the action result acceptor. Thus, actions, decisions and images of results can be adjusted during the implementation of the behavioral act in accordance with the available opportunities for its realization. Notably, FSs are “dimensionless”: they can encompass behavioral acts of different duration and scope, be nested within each other, and flow one into the other.

It can be seen that Anokhin's FST operates with abstract concepts describing any behavior as a whole, without reference to psychological or physiological processes, but capturing both psychological and physiological processes in a single united system. According to the theory, the components of the FS cannot be localized in specific organs or brain areas, but they are dynamically built during the realization of behavior based on the functional predispositions of organs and brain areas. Continual activity of the organism can reflect the dynamics of the FS.

Psychologically and physiologically, FST provides, first, a new perspective on behavior (with determination through goal and future outcome rather than past stimulation), and second, a possible solution to the brain-mind problem (mental processes cannot be localized in the brain, but components of the FS directed to the realization of behavioral acts can be observed using neuroimage methods). It can be observed that the FST shares conceptual similarities with other “active” neurocognitive theories. For instance, the predictive coding approach explains behavioral and brain activity by means of the continuous generation of hypotheses regarding the environment and their subsequent verification (Friston and Kiebel, 2009; Shipp, 2016). Additionally, both theories highlight the importance of predictions in brain activity and behavior, as well as the significance of error correction in this process. However, FST incorporates both physiological and psychological elements, combining them into a unified, irreducible system, while the predictive coding approach is better grounded into neurophysiological processes.

It is important to note that FST is a high-order abstract theory which describes holistic processes related to the interactions between biological organisms and the environment. Neural tracking, on the contrary, is an empirical phenomenon of electromagnetic brain activity. We believe that the FST framework can be applied to interpretations of neural tracking research results since it is already implemented in some neuroscience studies of cognition, perception, learning and emotions (Alexandrov and Sams, 2005; Alexandrov, 2015; Alexandrov and Pletnikov, 2022), it has rarely been compared with current trends in experimental studies. However, it can offer new perspectives on the planning and analysis of experiments.

## 3 A potential interpretation of neural tracking of speech using FST

It is noteworthy to briefly discuss that neural tracking of speech can be thought of as a part of an active process of speech comprehension. Indeed, usually studies provide active tasks to human participants (e.g., “please listen to the audio to further answer questions about its content”). In such task-setting a person is given a task with the final goal of answering questions, and the person's activity during the listening session will be determined by this goal (however, if the person has the internal or external motivation to complete the task). This is supported by studies of dichotic listening, in which only attended stimulus shows its neural tracking (Crosse et al., 2016; Broderick et al., 2018). The task of listening to only one channel determines the specific alignment of physiological systems, which in turn also determines where attention will be directed.

Thus, neural tracking can be represented as a *physiological part* of task-specific FS aimed at speech comprehension (Figure 1B). It can be assumed that in order to achieve the goal of speech comprehension, bottom-up components of FS are formed, aimed at decoding linguistic information from the acoustic speech signal, parsing linguistic units, their binding into higher order meaningful units (words, word combinations, phrases, sentences, narratives), and mapping these units to corresponding semantic content (Ding et al., 2016). It is essential that at this stage, a person's individual attitude to the listened speech is also developed, and emotional responses are formed. This can be attributed to the units of afferent synthesis and action (which has perhaps implicit nature) to perceive the linguistic unit that is now the focus of attention.

It is also known that speech perception is based on the principle of predictive coding of further content of speech (Hovsepyan et al., 2020; Heilbron et al., 2022); this can be attributed to the action result acceptor. The effects of semantic mismatch expressed in negative brain activity in response to the mismatch (the so-called N400 effect (Broderick et al., 2018; Nour Eddine et al., 2024)) can be interpreted as a correction of the action result acceptor via feedback afferentation. In this way, top-down regulation of neural tracking of speech based on its content and changes in this content can occur, which is confirmed by some studies (Broderick et al., 2019; Klimovich-Gray et al., 2023). In the structure of FS, this can be referred to action result acceptor, loops of efferent feedback, orienting feedback and decision-making (implicit decision-making about the congruence of the perceived and expected).

Interestingly, some EEG studies of speech perception show that pre-stimulus (before a sound or a word presentation) activity correlates with and predicts post-stimulus activity (Barraza et al., 2016; Hansen et al., 2019; Liu and Liu, 2025). We would like to propose that this phenomenon could be an indicator of the FS functioning through mechanisms of efferent and orienting feedback, and also the correction of the action results acceptor state.

In general, the outcome and positive adaptive effect of the considered FS is speech understanding at the psychological level and neural tracking at the physiological level.

## 4 Future directions

Why could FST be useful to interpret findings of neural tracking of speech studies? It is a theoretical framework that allows us to consider the psychological and physiological sides of cognitive processes as a whole. It allows these processes to be viewed as active, aimed at achieving an adaptive beneficial outcome. It is likely that this interpretation can help in furthering our understanding of brain functioning in different conditions, as well as in understanding the relationship between physiological and psychological processes.

Considering neural tracking of speech as an active process of speech comprehension raises new questions for empirical testing. How do experimental task peculiarities affect not only behavioral but also physiological outcomes? How can the content of speech stimuli and a person's attitude toward them influence neural tracking? What psychological factors, other than attention level, influence neural tracking?

Another line of work that is spawned by the FST approach is changing approaches to data analysis. According to the theory, system components cannot be localized to specific brain regions. It can be useful to apply network analysis methods and analyze global brain activity and its dynamics when listening to speech stimuli. It may be also of interest to investigate the relationship between pre-stimulus and post-stimulus EEG activity in response to words in the context of natural speech perception. Additionally, new predictive linguistic measures can be developed to analyze neural tracking of natural speech data. Current and widely used measures (e.g., semantic dissimilarity or word frequency) reflect the processing of words that have already been heard or are currently being heard; new measures should consider a subject's expectations regarding the upcoming content of speech (e.g., emotional attitudes, perception of the entire narrative, etc.).

The interpretation of neural tracking using FST has certain methodological limitations. While the key components of FST mentioned above do not necessarily have unambiguous neurobiological counterparts, their neurophysiological foundation requires further empirical verification. Additionally, it is necessary to investigate the extent to which neural tracking can truly be linked to the goal of the system. This can be achieved through experiments that involve goal-setting inconsistencies. Furthermore, FST offers a qualitative rather than quantitative description of processes, necessitating the development of mathematical tools. However, despite these limitations, the heuristic significance of FST should not be disregarded. Its emphasis on purposeful and flexible nature provides a framework for exploring the context-specific dynamics of neural tracking that other models may overlook.
